# Upper limb activity in myoelectric prosthesis users is biased towards the intact limb and appears unrelated to goal-directed task performance

**DOI:** 10.1038/s41598-018-29503-6

**Published:** 2018-07-23

**Authors:** A. Chadwell, L. Kenney, M. H. Granat, S. Thies, J. Head, A. Galpin, R. Baker, J. Kulkarni

**Affiliations:** 1 Centre for Health Sciences Research, University of Salford, Salford, Greater Manchester England; 2 Salford Business School, University of Salford, Salford, Greater Manchester England; 3 University Hospital of South Manchester NHS Foundation Trust, Withington, Greater Manchester England

**Keywords:** Outcomes research, Occupational health

## Abstract

Studies of the effectiveness of prosthetic hands involve assessing user performance on functional tasks, typically collected in the lab, sometimes combined with self-report of real-world use. In this paper we compare real-world upper limb activity between a group of 20 myoelectric prosthesis users and 20 anatomically intact adults. Activity was measured from wrist-worn accelerometers over a 7-day period. The temporal patterns in upper limb activity are presented and the balance of activity between the two limbs quantified. We also evaluated the prosthesis users’ performance on a goal-directed task, characterised using measures including task success rate, completion time, gaze behaviour patterns, and kinematics (e.g. variability and patterns in hand aperture). Prosthesis users were heavily reliant on their intact limb during everyday life, in contrast to anatomically intact adults who demonstrated similar reliance on both upper limbs. There was no significant correlation between the amount of time a prosthesis was worn and reliance on the intact limb, and there was no significant correlation between either of these measures and any of the assessed kinematic and gaze-related measures of performance. We found participants who had been prescribed a prosthesis for longer to demonstrate more symmetry in their overall upper limb activity, although this was not reflected in the symmetry of unilateral limb use. With the exception of previously published case studies, this is the first report of real world upper limb activity in myoelectric prosthesis users and confirms the widely held belief that users are heavily reliant on their intact limb.

## Introduction

Over-reliance on one upper limb may lead to overuse injuries^1–3^. Up to 65% of people with upper limb absence experience musculoskeletal complaints, compared to 34% of individuals without upper limb absence^4^. For a person with upper limb absence, one of the aims of prescription of a prosthesis is to restore a degree of function to the affected limb. In people with unilateral upper limb absence, a prosthesis which facilitates the execution of functional tasks may reduce the over-reliance on the anatomically intact side and this may, in turn, be reflected in upper limb activity patterns which are closer to those seen in anatomically intact individuals.

Self-report questionnaires are often used to elicit data on usage of prostheses in the real world. There is evidence that health questionnaires can be subject to bias and recall errors^5^, and provide, at best, only averaged data on daily wear and usage^6^. Previously^7^, building on a technique introduced by Bailey *et al*.^8^ to study upper limb activity in people with impairments following stroke, we introduced the use of wrist-worn activity monitoring sensors for the objective assessment of the upper limb activity of prosthesis users. The approach was illustrated with data collected from two trans-radial myoelectric prosthesis users and one anatomically intact participant, showing that in contrast to the data from the anatomically intact participant, the upper limb activity in both prosthesis users was heavily skewed towards their anatomically intact limb. However, it was not known whether these patterns of upper limb activity reported in Chadwell *et al*.^7^. are seen in larger groups of myoelectric prosthesis users and anatomically intact participants.

One question that arises from these observations is the strength of the relationship between impaired functioning of the upper limb and patterns of upper limb activity. Previously, Bailey *et al*.^8^ studied a population with unilateral arm impairments following stroke, and showed that performance on a validated measure of functionality (specifically the Action Research Arm Test – ARAT) did not strongly correlate with the real-world ‘*usage*’ of the affected arm; no studies have explored this, or related questions in a prosthesis user population. A number of recent studies have shown that performance on simple goal-directed tasks, evaluated using a combination of objective outcome measures, including task completion time, kinematics and gaze behaviour, provides a useful picture of the abilities of prosthesis users^9–13^. However, no studies have explored relationships between measures such as these and real world prosthesis use.

The main aim of this study was to report data collected over a 7-day period describing the upper limb activity of twenty people with trans-radial upper limb absence who have been provided with a myoelectric prosthesis and twenty anatomically intact participants. Using our novel approach to visualising the temporal patterns in upper limb activity data^6^ we also address a secondary research question, to investigate the extent to which previous self-report approaches, such as the Trinity Amputation and Prosthesis Experience Scales (TAPES)^14^ are capturing the true real-world patterns of upper limb activities. Finally, we investigate whether kinematic and gaze-related measures of performance on a multi-stage goal-directed task correlate with different measures of upper limb activity (‘*usage*’ measures) in the same group of twenty people with upper limb absence who have been provided with a myoelectric prosthesis.

## Methods

### Participants

Twenty participants (14 male, 6 female) with unilateral upper limb absence at a trans-radial level were recruited from six (4 NHS, 2 University) sites across the UK. All participants had a single degree of freedom myoelectric prosthesis (e.g. Steeper Select or Ottobock DMC Plus/VariPlus/Sensor Speed).

The age of the prosthesis users ranged from 18 to 75 years (mean age 53 years). Eleven people had congenital limb absence (6 Right/5 Left), and nine had an amputation (6 Right/3 Left); six of the amputations had occurred on the dominant side. Time since amputation ranged from 8–47 years (mean 25 years). Time since prescription of a myoelectric prosthesis ranged from 1.5–39 years (mean 20 years).

A group of twenty anatomically intact participants (9 male, 11 female, age 23–61, mean age 43, 3 left handed) with no upper limb impairments were also recruited through the University of Salford.

Ethical approval for this study was granted by the University of Salford School of Health Sciences Research Ethics committee (REF: HSCR 16–25), by the University of Strathclyde Department of Biomedical Engineering Ethics Committee (DEC.BioMed.2017.220) and through the NHS IRAS system (IRAS Project ID: 193794). Informed consent was gained from all participants.

### Equipment

To evaluate upper limb activity in the real world, we used Actigraph activity monitoring sensors from the GT3X range (GT3X+, wGT3X, wGT3X-BT). These sensors provided continuous logging of acceleration across three axes at 30 Hz.

Prosthesis user goal-directed task performance was assessed during performance of a multistage task (“*cylinder task*”)^7^. An electronic goniometer (Biometrics Ltd) was attached across the proximal knuckle of the index finger (on the prosthetic hand) to measure hand aperture. An Inertial Measurement Unit (IMU) (Xsens MTw) was fixed on the forearm to measure forearm motion. A head-mounted eye tracker (Dikablis Professional Wireless) was worn to capture gaze behaviour. A button was placed beneath the hand in the starting position for synchronisation purposes. Full details of the “*cylinder task*” and the assessment measures are provided in our previous paper^7^.

### Protocol

The methods for the assessment of everyday activity used in this study are described in Prosthetics and Orthotics International^6^, however, since this publication a couple of amendments were made to the protocol. A newer version of the Actilife software was used (Actilife6 for compatibility with the newer sensors) and an updated non-wear algorithm was developed as detailed in the supplementary material.

#### Data Collection – Everyday Activity

The sensors were initialised using Actilife6 software and programmed to record data for 7 days at 30 Hz. The start time was set so that the participant was wearing the sensors at the onset of data recording.

Participants were asked to wear the sensors, one on each wrist, for a 7-day period. The monitors were labelled to indicate on which wrist, and in which orientation they should be worn. Participants were requested to remove the monitors, only when they may become wet. As the myoelectric prosthesis would not be worn during bathing or showering, participants were instructed to leave the prosthesis-worn monitor on throughout the testing period.

Participants were asked not to alter their behaviour during the data collection period and to keep a simple diary, which would be used to assist with the interpretation of the data. This diary included the recording of sleep/wake times, periods of sensor removal, and for the prosthesis users, periods of prosthesis removal.

At the end of the 7-day period, participants were asked to return the sensors and the completed diary either in person or by post.

#### Data Collection – Goal Directed Task Performance

To evaluate the prosthesis user’s goal-directed task performance, they were asked to reach to grasp a cylinder, lift and rotate it 90 degrees to the horizontal, and place it inside a tube^7^ (“*cylinder task*”). Participants were asked to attempt the task ten times.

Task onset (the start of reach to grasp) was defined as the onset of movement (either lifting the arm or opening the hand), the end of reach to grasp was defined as the point at which the fingers finished closing around the cylinder, and task completion was defined as the moment the fingers began to open to release the cylinder after it had been placed into the tube.

It is worth noting that data collection for this part of the task was undertaken at the participant’s local prosthetics clinic, using our portable experimental equipment.

#### Activity Data Preparation Proprietary To Actilife Software

Data were downloaded using Actilife6 software. A low frequency extension filter (proprietary to the Actilife software) was employed^15^. The filtered accelerations were grouped into one minute epochs and converted into activity counts (for each of the three axes) using proprietary algorithms^16^. For each epoch, the resultant of the activity counts across the three axes was calculated generating the Vector Magnitude (VM). The VMs were exported to MATLAB (v. 2016a) for further analysis.

#### Limb Dominance Terminology

As this paper reports data from both anatomically intact participants and persons with upper limb absence who have been prescribed a myoelectric prosthesis, we define the terminology used to describe the limbs as follows.

The upper limb of each anatomically intact subject with which they self-reported to write was defined as the dominant, with their other limb being the non-dominant.

To reduce the number of equations used to characterise upper limb activity, we use the same variable names when referring to both anatomically intact participants and the prosthesis users. Therefore, we label both the anatomically intact upper limb of participants with unilateral upper limb absence and the dominant limb of the anatomically intact participants as the dominant limb; we label the other limb as the non-dominant limb^17^. However, within the text of the Results and Discussion sections, we refer to the **anatomical arm** and the **prosthesis** for ease of understanding.

#### Removal Of Prosthesis Non-Wear Time

As we were interested in how the prosthesis was used during the periods when it was worn, a method of removing the non-wear periods was required. “*Prosthesis non-wear*” was assumed to correspond to the times when the monitor worn on the wrist of the prosthesis recorded prolonged inactivity. In our earlier paper^7^ we developed an algorithm for removal of these “*prosthesis non-wear*” periods, which required some visual inspection of the data. Subsequently a more automated algorithm for the removal of “*prosthesis non-wear*” periods was developed (see the supplementary material).

#### Data Analysis – Everyday Activity

For the prosthesis users we calculated the amount of time (in hours) spent wearing the myoelectric prosthesis over the 7-day period. This is referred to as “***Prosthesis wear time (C)***” calculated by subtracting the “*prosthesis non-wear*” periods from the overall recording time. We use the letter C in parentheses to distinguish wear time calculated using the non-wear algorithm “***Prosthesis wear time (C)***” from self-reported wear time “***Prosthesis wear time (SR)***”.

For both cohorts we also calculated the balance of activity across both limbs. First, for every 1-minute epoch, the percentage reliance on the dominant side (“***% Reliance***_***Dom***_”) was calculated based on the VM values from the sensors on each arm. Where the VM was **equal to 0** on both arms, the epoch was marked as ‘**both arms at rest**’. For all other epochs the VM on the dominant side was divided by the sum of the VM across both arms to calculate the percentage reliance on the dominant side:

$$[ \% {Relianc}{{e}}_{{Dom}}\,=\,{round}({V}{{M}}_{{Dom}}/({V}{{M}}_{{Dom}}+{V}{{M}}_{{NonDom}})\times 100)]$$

Two summary measures were calculated to characterise the balance of activity, one considering all data during which either or both arms were moving (“***Median %Reliance***_***Dom***_”), and one considering only the data during which activity was seen on only one limb (“***Unilateral ratio***”). Epochs previously marked as ‘**both arms at rest**’ were excluded from this analysis. For prosthesis users, “*prosthesis non-wear*” periods (calculated using the non-wear algorithm) were also excluded.

1. “***Median %Reliance***_***Dom***_” defined as the median of all of the “***%Reliance***_***Dom***_” values.
2. “***Unilateral ratio***” defined as the ratio between the unilateral activity on the dominant and non-dominant sides (“***UL***_***Dom***_”: “***UL***_***NonDom***_”). Here, unilateral dominant activity (“***UL***_***Dom***_”), was defined as the number of minutes where activity counts were only recorded on the sensor on the dominant limb (VM_NonDom_ = 0); Unilateral non-dominant activity (“***UL***_***NonDom***_”) was defined as the number of minutes where activity counts were only recorded on the non-dominant limb sensor (VM_Dom_ = 0).

#### Data Analysis – Goal Directed Task Performance

For each participant, performance of the “*cylinder task*”, was evaluated using a series of previously reported measures:

1. **Task success** – The total number of successful trials (out of 10). Trials where the movement was not smooth (e.g. the hand opened and closed more than once during reach-to-grasp), and trials where the cylinder was not placed all of the way into the tube (but remained in place once released) were counted as successful but only scored half a point. Trials where the cylinder was knocked over or dropped were counted as unsuccessful.
2. **Task duration** – The mean duration of the successful attempts (in seconds).
3. **“Delay plateau”** – Bouwsema^13^ found that prosthesis users demonstrate a delay in the onset of hand opening at the start of reach-to-grasp not generally seen in anatomically intact subjects. For those trials where the user successfully achieved a grasp, the time between the task onset (identified using the IMU/goniometer) and the onset of hand opening (identified using the goniometer) was calculated and expressed as a percentage of reach-to-grasp (completion identified using the goniometer). A mean value was reported for each participant.
4. **“Reach plateau”** – Bouwsema^12^ also found that prosthesis users demonstrate a characteristic plateau in their hand aperture during reach-to-grasp. The length of this plateau was shown to reduce with improved functionality (measured using the Southampton Hand Assessment procedure). For those trials where the user successfully achieved a grasp, we identified the plateau periods based on the hand aperture being within two degrees of the maximum (using the goniometer data), and calculated the durations as a percentage of reach-to-grasp. A mean value was reported for each participant.
5. **Acceleration temporal variability** – Prosthesis users have been shown to demonstrate increased trial-trial temporal variability in the trajectories of wrist-worn accelerometer data when compared to anatomically intact subjects^18^. Furthermore, this variability has been shown to decrease with practice^19^. Here we assessed the temporal variability in the acceleration of the forearm between successful trials (calculated according to the methods of Thies *et al*.^20^ using the IMU).
6. **Gaze patterns** – Prosthesis users have been shown to spend a proportion of the reach-to-grasp phase focussing on their hand and/or the area of the object to be grasped (grasp critical area or GCA); by contrast anatomically intact participants generally look ahead to plan subsequent parts of the task, rarely looking at their hand or the GCA^21,22^. During the transport phase, similar patterns of behaviour are seen, with prosthesis users focusing on the hand and/or GCA. Here we report:
  The % of the reach-to-grasp phase spent looking at:
    the hand
    the grasp critical area (GCA bottom half) of the cylinder
    the location critical area (LCA top half) of the cylinder, or the tube
  The % of the transport phase spent looking at:
    the hand or the GCA of the cylinder
    the LCA of the cylinder, or the tube

#### Data Analysis - Correlations

Due to the size of the dataset and some measures containing a large number of tied ranks (e.g. success rate), Kendall’s Tau-b (2-tailed) was used to establish whether any significant correlations existed between the goal-directed task performance and the everyday upper limb activity measures. Analysis was undertaken using IBM SPSS Statistics software v24.0.0.1.

#### Data Visualisation – Everyday Activity

Two types of data visualisation were used to display the activity data. Archimedean spiral plots^6^ were used to illustrate temporal patterns in the upper limb activity, and histograms were produced to characterise the distribution of activity between the two upper limbs.

**Figure 1:**
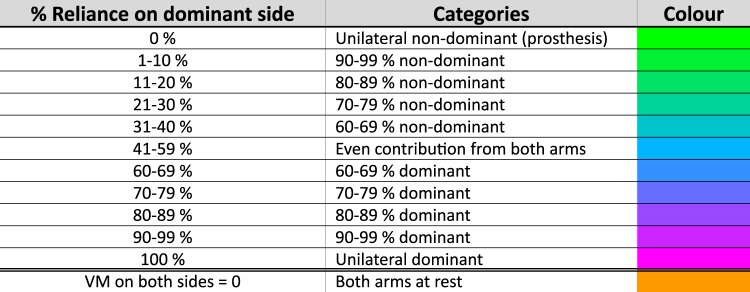
Allocation of VM data (per epoch) into categories.

To generate the spiral plots the “***%Reliance***_***Dom***_” values for each epoch were categorised according to the values in Fig. 1. The colour coded data were plotted using the spiral timeseries visualisation introduced in our earlier paper^6^. Working outwards, each revolution signified 24 hours, with midnight at the top and midday at the bottom.

A histogram of the “***%Reliance***_***Dom***_” values for each epoch was also produced. The data were grouped into activity bins (in 1% increments), and the number of minutes of data within each bin was plotted on the y-axis. For ease of visualisation, the time was displayed on a log_10_ scale.

N.B. “*Prosthesis non-wear*” periods (according to the non-wear algorithm) and times when both arms were at rest were not included in the histogram.

### Data availability

The datasets generated during the study are available from the corresponding author on reasonable request.

## Results

### Everyday upper limb activity of myoelectric prosthesis users

In this section the results of the analysis for the twenty myoelectric prosthesis users are presented. It is important that we distinguish between the amount of time the prosthesis was worn during the week (“***prosthesis wear time (C)***”) and the actual ‘*usage*’ of the prosthetic arm (quantified using “***Median %Reliance***_*Dom*_”) during these periods; these two points are therefore addressed separately.

#### Self-Reported Prosthesis Wear Time

The quality of the self-report data differed between subjects. Five participants failed to report a full set of times for the removal of the prosthesis and/or monitors (for example the participants would self-report the prosthesis to be removed, but not report it being put back on); additionally, one participant did not complete the diary at all and instead provided a written account of their non-wear from memory.

For the 14 remaining prosthesis users, the self-reported “***prosthesis wear time (SR)***” was compared to the calculated “***prosthesis wear time (C)***”. On average (median) the algorithm calculated the “***prosthesis wear time (C)***” over the 7 days to be 4.4 hours shorter than self-reported (Maximum negative difference = −52.6 hours (calculated shorter), maximum positive difference = 6.3 hours (calculated longer), Q1 = −9.5 hours, Q3 = 0.8 hours). Approximately 35% of participants showed a difference between self-reported and calculated wear times of less than 5% of the total “***prosthesis wear time (C)***”.

For all subsequent analysis, the “*prosthesis non-wear*” periods were removed using the non-wear algorithm.

#### Prosthesis Wear Time Calculated Using The Non-Wear Algorithm

**Figure 2:**
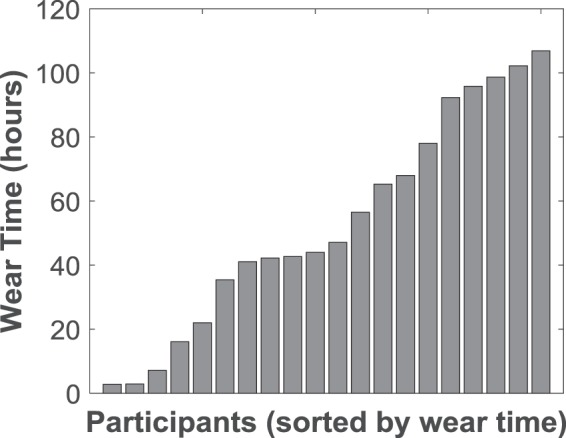
“***Prosthesis wear time (C)***” as calculated using the non-wear algorithm for each of the 20 participants. Median = 45.6 hours (IQR = 63.4).

Five of the participants wore the prosthesis all day, every day, removing it only to sleep (“***prosthesis wear time (C)***” > 91 hours / 13 hours per day, maximum = 106.9 hours per week). Two participants wore the prosthesis for less than 3 hours over the 7 day period (minimum “***prosthesis wear time (C)***” = 2.8 hours per week). The remainder of the participants either wore the prosthesis during the daytime removing it in the evenings each day, altered their wear pattern throughout the week, or wore the prosthesis only for short periods. The median “***prosthesis wear time (C)***” was 45.6 hours per week (Q1 = 25.4, Q3 = 88.7) (Fig. 2).

#### Prosthesis Usage

The primary measure of ‘*prosthesis usage*’ was the “***Median %Reliance***_***Dom***_”. For each participant this median value was calculated based only on the times the prosthesis was worn. Histograms were plotted based on the percentage reliance on the anatomically intact side for each minute of data collected (50% signifying an equal Vector Magnitude recorded on each sensor for that minute).

**Figure 3:**
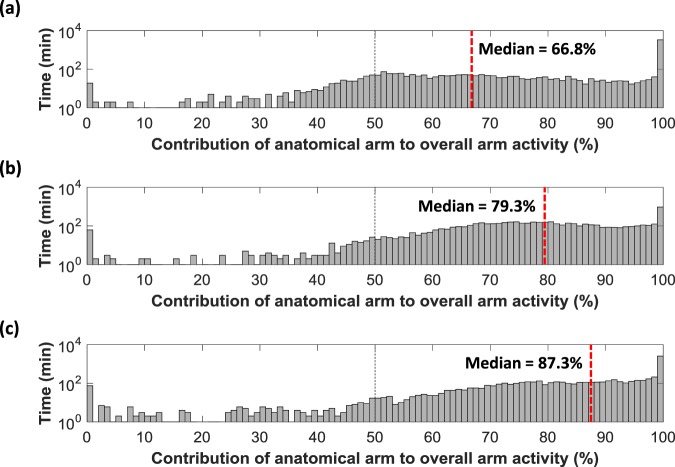
Histograms for the participants with (**a**) the lowest “***Median %Reliance***_**Dom**_” value, (**b**) the “***Median %Reliance***_**Dom**_” value closest to the mean and median of all twenty participants, and (**c**) the highest “*Median %Reliance*_*Dom*_” value (the person most reliant on their anatomical arm). NB. The time (y-axis) is displayed using a log_10_ scale to mitigate for large amounts of unilateral activity.

All of the prosthesis users demonstrated a skew in the histogram towards the anatomical side (>50%). This was supported by “***Median %Reliance***_***Dom***_” values ranging from 66.8% up to 87.3% reliance on the anatomical side (median = 79.9%, Q1 = 74.5%, Q3 = 84.7%). Figure 3 presents example histograms for three of the participants: **(A)** the person least reliant on the anatomical side, **(B)** a person from the middle of the dataset, and **(C)** the person most reliant on the anatomical side.

We found there to be a medium negative correlation between time since prescription of a myoelectric prosthesis and the “***Median %Reliance***_***Dom***_” (Kendall’s τ_b_ = −0.464, p = 0.005, n = 20). No other correlations were found between demographic data (age, gender, cause of limb absence, time since limb loss, time since prescription of a myoelectric prosthesis, side of limb absence, dominance pre limb loss) and any of the other outcome measures.

A secondary measure of ‘*prosthesis usage*’ was the “***unilateral ratio***”, defined as the ratio between unilateral activity on the dominant and non-dominant sides. The time spent using the anatomical arm alone (the bar at 100%) was higher than the unilateral use of the prosthesis (the bar at 0%) for all participants. The minimum “***unilateral ratio***” was 4.3 minutes of unilateral use of the anatomical side for each minute spent using the prosthesis in a unilateral manner (356 mins anatomical, 82 mins prosthesis, “***prosthesis wear time (C)***” = 98.7 hours). Two participants demonstrated 0 minutes of unilateral prosthesis use resulting in an undefined “***unilateral ratio***”. For the remaining eighteen participants, the “***unilateral ratios***” of “***UL***_***Dom***_”: “***UL***_***NonDom***_” were as follows: minimum = 4.3:1, first quartile = 9.6:1, median = 11.5:1, third quartile = 21.8:1, and maximum = 73:1.

#### Prosthesis Wear Time Vs Prosthesis Usage

**Figure 4:**
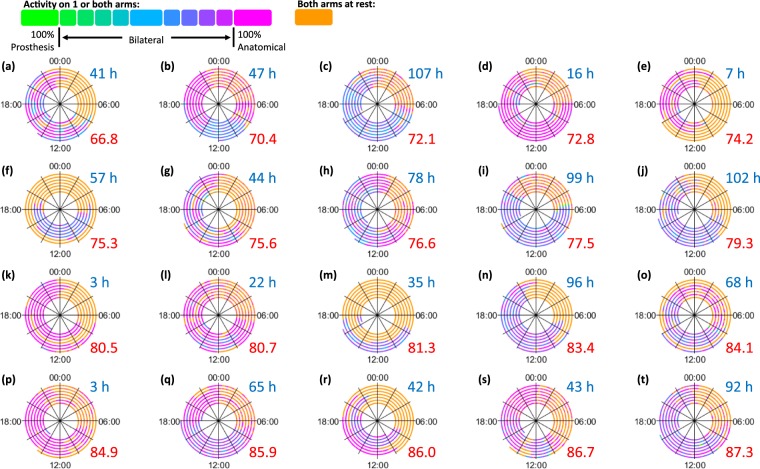
Spiral plots for all 20 myoelectric prosthesis users. The plots are ordered according the “***Median %Reliance***_***Dom***_” values (calculated based on the times the prosthesis was worn) from the person who is the least reliant on the anatomical side to the person who is the most reliant. The “***prosthesis wear time (C)***” for each user is shown in blue (rounded to the nearest hour), and the “***Median %Reliance***_*Dom*_” is shown in red.

It is important to note that increased “***prosthesis wear time (C)***” does not necessarily correspond to a more symmetrical arm ‘*usage*’ pattern during the times when the prosthesis was actually worn (Kendall’s τ_b_ = 0.032, p = 0.846, n = 20). Figure 4 shows the spiral plots for all twenty prosthesis users ordered according to the “***Median %Reliance***_***Dom***_” values (shown in red), calculated based only on the data from the times when the prosthesis was worn; the associated “***prosthesis wear time (C)***” is also reported (shown in blue and rounded to the nearest hour).

The five ‘all-day wearers’ (“***prosthesis wear time (C)***” >91 hours) are actually spread throughout the group (Fig. 4c,i,j,n,t); whilst the person with the most symmetrical arm ‘*usage*’ (Fig. 4a, “***Median %Reliance***_***Dom***_” =66.8% reliance on the anatomical arm) donned and doffed the prosthesis regularly throughout the day (“***prosthesis wear time (C)***” = 41 hours).

### Upper limb activity of anatomically intact participants

Most anatomically intact participants were slightly more reliant on their dominant side (“***Median %Reliance***_***Dom***_” >50%), whilst some showed a slight preference towards their non-dominant side (“***Median %Reliance***_***Dom***_” <50%). Across the twenty participants, the “***Median %Reliance***_***Dom***_” values ranged from 43.9% to 62.8% (median = 51.3%, Q1 = 49.3%, Q3 = 53.6%).

The anatomically intact subjects showed a high frequency of unilateral activity at 0% (unilateral non-dominant) and 100% (unilateral dominant). The height of these bars were similar on both sides of the histogram; the “***unilateral ratio***” of “***UL***_***Dom***_”: “***UL***_***NonDom***_” can be described as follows: minimum = 0.42:1, first quartile = 0.79:1, median = 1.31:1, third quartile = 1.74:1, and maximum = 2.08:1.

### Comparing the upper limb activity of prosthesis users to the anatomically intact participants

**Figure 5:**
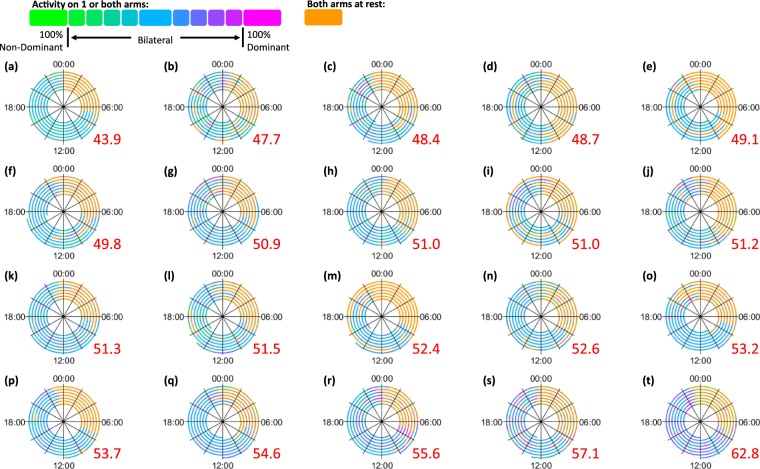
Spiral plots for all 20 anatomically intact subjects. The plots are ordered according the “***Median %Reliance***_*Dom*_” values from the person who is the least reliant on the dominant side to the person who is the most reliant. The “***Median %Reliance***_***Dom***_” is shown in red.

Figure 5 shows the spiral plots for all twenty anatomically intact participants. An immediate colour difference can be seen when comparing these plots to the spirals for the prosthesis users in Fig. 4. The spirals for the anatomically intact subjects tend to be primarily blue, with portions of both green and magenta corresponding to activities where each arm is used in a unilateral manner. The spirals for the prosthesis users tend to be purple, with large portions of magenta (corresponding to a preference towards the anatomical arm), and very little green (which would correspond to a preference towards the prosthesis).

**Figure 6:**
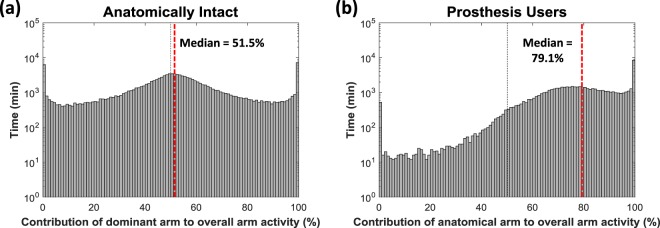
Histograms showing the grouped data for (**a**) all 20 anatomically intact subjects and (**b**) all 20 prosthesis users. The anatomically intact participants are similarly reliant on both arms (“***Median %Reliance***_***Dom***_” =51.5%), whilst the prosthesis users are more reliant on the anatomically intact side (“***Median %Reliance***_***Dom***_” =79.1%).

To provide an overview of this ‘*usage*’ data, in Fig. 6a the data recorded for all twenty anatomically intact subjects is grouped into a single histogram with an overall “***Median %Reliance***_***Dom***_” value of 51.5%. When comparing this group histogram to the grouped data recorded from the twenty prosthesis users (Fig. 6b) (“***Median %Reliance***_***Dom***_” = 79.1%), a clear difference in the shape of the histogram can be seen. As noted previously, the prosthesis users are heavily reliant on the anatomically intact arm, and periods where more activity occurs on the prosthetic side than on the anatomical side (% contribution < 50%) are comparatively rare (≈18 min > 50% for each minute ≤50% compared to ≈1:1 in the anatomically intact group).

It is worth noting that for the prosthesis users, the data included in the histogram and “***Median %Reliance***_***Dom***_” calculations are only from the times when the prosthesis was worn, consequently the overall number of data points was lower than that used in the calculations for the anatomically intact subjects.

**Figure 7:**
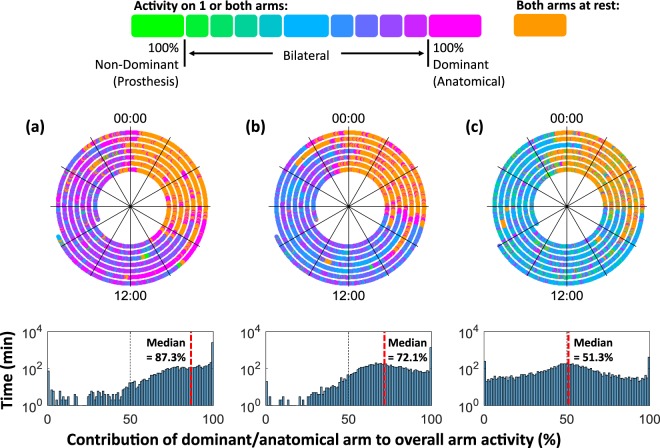
(**a**) The all-day prosthesis wearer with the highest “***Median %Reliance***_***Dom***_” value (=87.3%), (**b**) the all-day wearer with the lowest “***Median %Reliance***_***Dom***_” value (=72.1%), and (**C**) an average anatomically intact participant (“***Median %Reliance***_***Dom***_” =51.3%).

To illustrate the differences between prosthesis users, Fig. 7 presents data from two prosthesis users who wore the prosthesis all day every day, and for comparison, an average anatomically intact participant. Both prosthesis users demonstrated a skew in the histogram with a preference towards their unaffected arm, however the participant in Fig. 7b showed more pronounced curvature than the participant in Fig. 7a with a peak around 65–75%. As participants use their prosthesis more, this peak would be expected to shift towards the centre as seen in the anatomically intact example (Fig. 7c).

### Correlations between goal-directed task performance and everyday upper limb activity

Table 1 presents the results of the Kendall’s Tau-b correlations between the measures of goal-directed task performance (incl. task success, duration and kinematic and gaze based measures of performance) and the measures of everyday upper limb activity (signifying prosthesis wear and ‘*usage*’) for the twenty prosthesis users. No significant correlations (p < 0.05) were found between any of the measures of goal-directed task performance and the measures of everyday activity evaluated using the activity monitors.

**Table 1:** Kendall**’**s Tau-b (2-tailed) correlations between the measures of goal-directed task performance and the measures of everyday activity. NB. The calibration of the eye tracker failed for 1 participant, and the “***Unilateral Ratio***” for 2 participants was undefined (no unilateral prosthesis activity); these participants were excluded from the relevant correlations (n = number of values included in correlation)

	Success Rate	Task Duration	Delay Plateau Length	Reach Plateau Length	Acceleration Variability	Reach-to-grasp %Hand	Reach-to-grasp %GCA	Reach-to-grasp %LCA/Tube	Transport %Hand/GCA	Transport %LCA/Tube
Median %Reliance Dominant	τ = 0.071 p = 0.681 n = 20	τ = 0.105 p = 0.516 n = 20	τ = 0.158 p = 0.330 n = 20	τ = −0.074 p = 0.650 n = 20	τ = −0.053 p = 0.746 n = 20	τ = 0.030 p = 0.860 n = 19	τ = 0.076 p = 0.649 n = 19	τ = −0.041 p = 0.807 n = 19	τ = −0.129 p = 0.441 n = 19	τ = 0.181 p = 0.278 n = 19
Unilateral Ratio	τ = 0.200 p = 0.272 n = 18	τ = −0.150 p = 0.384 n = 18	τ = 0.020 p = 0.910 n = 18	τ = 0.020 p = 0.910 n = 18	τ = −0.255 p = 0.140 n = 18	τ = −0.260 p = 0.155 n = 17	τ = −0.015 p = 0.934 n = 17	τ = 0.309 p = 0.084 n = 17	τ = −0.199 p = 0.266 n = 17	τ = 0.088 p = 0.621 n = 17
Prosthesis Wear Time	τ = 0.047 p = 0.784 n = 20	τ = 0.000 p = 1.000 n = 20	τ = 0.053 p = 0.746 n = 20	τ = −0.158 p = 0.330 n = 20	τ = −0.011 p = 0.948 n = 20	τ = 0.066 p = 0.697 n = 19	τ = 0.251 p = 0.132 n = 19	τ = −0.263 p = 0.115 n = 19	τ = −0.117 p = 0.484 n = 19	τ = 0.287 p = 0.086 n = 19
Unilateral Dominant	τ = 0.083 p = 0.632 n = 20	τ = 0.116 p = 0.475 n = 20	τ = 0.126 p = 0.436 n = 20	τ = −0.232 p = 0.153 n = 20	τ = 0.105 p = 0.516 n = 20	τ = −0.042 p = 0.805 n = 19	τ = 0.088 p = 0.600 n = 19	τ = −0.053 p = 0.753 n = 19	τ = −0.235 p = 0.161 n = 19	τ = 0.263 p = 0.115 n = 19
Unilateral Non-dominant	τ = −0.191 p = 0.273 n = 20	τ = 0.281 p = 0.085 n = 20	τ = 0.143 p = 0.380 n = 20	τ = −0.207 p = 0.205 n = 20	τ = 0.218 p = 0.183 n = 20	τ = 0.128 p = 0.457 n = 19	τ = 0.130 p = 0.441 n = 19	τ = −0.201 p = 0.233 n = 19	τ = −0.065 p = 0.700 n = 19	τ = 0.118 p = 0.483 n = 19

.

## Discussion

### Sample size

Although there are no national statistics on upper limb prosthesis provision in the UK, based on our clinical contacts we estimate that there are approximately 800–1000 myoelectric prosthesis users registered to NHS limb centres in the UK. This multi-site study of twenty people who have received a myoelectric prosthesis is one of the largest experimental studies of this population undertaken in the UK to date. We note that the prosthesis users in this study are on average 10 years older than the anatomically intact subjects; however, it has been shown that for healthy adults the number of hours of upper limb activity is not associated with age^23^. Therefore, the age difference between the groups was unlikely to have had a major impact on our findings.

### Prosthesis wear time

Prosthesis users ranged from people who rarely wore their prosthesis through to people who wore the prosthesis all day every day.

In this study self-report diaries were shown in some cases to have the potential to provide an extremely accurate measure of ‘*prosthesis wear time*’, however, the reliability of the person providing the data could not be guaranteed (in this study 30% of participants failed to provide completed diaries). Consequently automated algorithms based on the data from the activity monitors were used to report ‘*prosthesis wear time*’.

Until now the primary measure of prosthesis wear has been the average daily ‘*prosthesis wear time*’. For some participants, the wear patterns varied in a complex manner over time. For example, the participant represented in Fig. 4g demonstrated a highly variable wear pattern. On some days this participant wore the prosthesis for 9–11 hours, whilst on other days they chose to wear the prosthesis for less than 4 hours, or even not at all. Consequently, a single value constituting the average daily “***prosthesis wear time (C)***” would provide limited insight into the long-term wear pattern for this user.

Similarly, two users who exhibit the same average “***prosthesis wear time (C)***” may wear their prosthesis in a very different manner. For example, the two participants represented in Fig. 4a and r have weekly “***prosthesis wear times (C)***” of 41 and 42 hours respectively; nevertheless their wear patterns are visibly very different with one user regularly taking the prosthesis on and off, whilst the other wore it for the full day, but only on 4 of the days of testing.

The spiral plot time series visualisations provide context to the “***prosthesis wear time (C)***” to help understand the patterns of wear.

### Quantifying prosthesis usage

As mentioned in the introduction, it is reasonable to suppose that provision of a myoelectric prosthesis to a person with unilateral upper limb absence may lead to a lower reliance on their anatomically intact side. Not only does this mean that we should be evaluating whether the prosthesis is worn, but also the actual ‘*usage*’ of the prosthesis should be measured (no significant correlation (p < 0.05) was found between these measures).

The techniques used in this paper allow us to both visualise and quantify a prosthesis user’s progression towards the symmetrical upper limb use demonstrated by anatomically intact participants. Nevertheless, as can be seen in Fig. 7b, there is still a clearly visible difference between the prosthesis user who displayed the longest “***prosthesis wear time (C)***” combined with the highest level of ‘*prosthesis usage*’, and the anatomically intact example (Fig. 7c).

All of the prosthesis users demonstrated an increased reliance on their anatomically intact side. However, we noted that participants who had been prescribed a myoelectric prosthesis for longer tended to be less reliant on their anatomically intact side (“***Median %Reliance***_***Dom***_” closer to 50%). No correlations were seen between the demographic data and the other outcome measures, including the other measure of upper limb symmetry (the “***unilateral ratio***”). Further work would therefore be needed to establish the importance of this finding.

Previous work has suggested that a person with no upper limb impairments is equally reliant on both of their arms during daily life^6–8^ (with a very slight preference towards the dominant side equivalent to 52% reliance^8^). Similar to the findings of these other studies we found our anatomically intact participants to be evenly reliant on each of their arms (51% reliance on dominant side).

### The relationship between goal-directed task performance and everyday upper limb activity

This paper also addressed the secondary research question as to whether a relationship exists between user performance on a goal-directed task and their everyday upper limb activity. It has been established that the primary method of evaluating the ‘success’ of a prosthesis within the lab is through the use of assessments of user performance on manual tasks. Outcome measures often include task success and duration, or more recently within the lab measures such as gaze behaviour, kinematic variability or patterns in hand aperture.

In this study we found there to be no significant correlation (p < 0.05) between any of the lab-assessed measures of user performance on a goal-directed task and the measures of ‘*prosthesis wear*’ and ‘*usage*’. A simple assumption might be that the better a prosthesis user performs within the lab, the more likely they are to use the prosthesis to perform everyday tasks. However, we found no evidence linking our measures of user performance with usage, suggesting further studies are needed.

## Limitations and Future Work

Our previous paper^6^ highlighted some of the limitations with these methods for the assessment of upper limb prosthesis users outside of the clinic using activity monitors. Most notably, our current measure of ‘*prosthesis usage*’ does not account for the active use of the prosthetic hand (as opposed to its use to, for example, simply stabilise an object). Future studies should complement the wrist-worn accelerometer data with a log of activations of the hand. Spiers *et al*.^24^ recently used a head mounted camera to evaluate terminal device use outside of the clinic. Their study demonstrated that non-prehensile grasp occurs significantly more than prehensile grasp. These methods could be used to complement the data from activity monitors, however, their methods involve very time-consuming analysis and a more efficient method of evaluation of hand movements should be explored for use by clinicians.

It is also worth noting that at present the automated non-wear algorithm is not able to differentiate between the prosthesis or the monitors being carried and being worn, therefore, it is possible that the algorithm may provide a slight over-estimate of the ‘*prosthesis wear time*’. Furthermore, there is currently no way to determine whether the monitor has been removed from the wrist of the prosthesis; consequently if the prosthesis was worn but the sensors were not then this was counted as “*prosthesis non-wear*” (see the *supplementary material*). One participant self-reported removing the sensors from the prosthesis during one of the days of data collection. Further work (and potentially some additional sensors^25^) would be required to provide an entirely accurate measure of the “*prosthesis non-wear*” periods.

In the case of a person not demonstrating any minutes of unilateral prosthesis use over the recording period, the methods presented in this paper do not allow the calculation of the “***unilateral ratio***”. The two participants who showed no unilateral prosthesis use were observed to wear their prosthesis for very short periods over the 7-day monitoring period (“***prosthesis wear time (C)***” = 2.2 hours and 22 hours over the 7 days), which is perhaps unsurprising. It may be worthwhile in the future exploring alternative methods of representing unilateral activity.

In this paper we have begun to question the relationship between performance on a goal-directed task and upper limb activity outside the clinic. Our task was an extension of the task used by Bouwsema *et al*.^13^ and in common with many other tasks used to explore performance, involved a reach-to-grasp phase. The measures of performance used in this study are not widely used within clinical environments, therefore future studies may want to consider repeating this comparison using validated assessments of functionality such as the Southampton Hand Assessment Procedure (SHAP)^26^ or the clothespin relocation test^27^ to build on our finding of no clear correlations between our measures and real-world use of a prosthesis.

Activity monitoring offers a quick and easy way of evaluating actual ‘*prosthesis wear*’ and ‘*usage*’ outside of the clinical environment. Initialisation of the sensors takes around 5 minutes, and downloading the data/running our code takes around 5–10 minutes. Thus allowing the clinician to analyse the data in the presence of the patient as an aid to discussions. This information would be useful across the industry, from the development of new devices, to the commissioning and prescription processes, the evaluation of intervention effectiveness, and as part of the rehabilitation process. Through the further development of these measures, we have the opportunity to gather data from a large dataset of prosthesis users, expanding our understanding of the factors affecting everyday prosthesis use.

## Summary

In this paper we have introduced data exploring the everyday upper limb activity (using activity monitors) of twenty myoelectric prosthesis users, and compared these to twenty adults with no upper limb impairment.

We have demonstrated that longer “***prosthesis wear time (C)***” (the most common method of assessing how a person uses their prosthesis outside of the clinic), does not necessarily correspond to greater ‘*usage*’ of the prosthesis relative to the anatomically intact arm (quantified based on the “***Median %Reliance***_***Dom***_”). We found participants who had been prescribed a prosthesis for longer to be more symmetrical in their upper limb activity according to the “***Median %Reliance***_***Dom***_” (τ_b_ = −0.464, p = 0.005, n = 20), however, no significant correlation (p < 0.05) was seen with the “***Unilateral ratio***”. We found no significant correlations (p < 0.05) between kinematic and gaze-related measures of performance on the goal-directed task and our measures of everyday upper limb activity.

We conclude that our methods using activity monitoring sensors offer a more objective and accurate outcome measure for the assessment of prosthesis user performance outside of the clinic. We suggest that further work is needed to enhance these outcome measures, and to increase the size of the dataset to develop standards for the representation of data on real-world upper limb activity.

## Electronic supplementary material

Supplementary Material

## References

[1] Gambrell CR (2008). Overuse syndrome and the unilateral upper limb amputee: consequences and prevention. JPO: Journal of Prosthetics and Orthotics.

[2] Jones LE, Davidson JH (1999). Save that arm: A study of problems in the remaining arm of unilateral upper limb amputees. Prosthet. Orthot. Int..

[3] Østlie K, Franklin RJ, Skjeldal OH, Skrondal A, Magnus P (2011). Musculoskeletal pain and overuse syndromes in adult acquired major upper-limb amputees. Archives of Physical Medicine and Rehabilitation.

[4] Postema SG (2016). Musculoskeletal Complaints in Transverse Upper Limb Reduction Deficiency and Amputation in The Netherlands: Prevalence, Predictors, and Effect on Health. Archives of Physical Medicine and Rehabilitation.

[5] Choi BCK, Pak AWP (2005). A Catalog of Biases in Questionnaires. Preventing Chronic Disease.

[6] Chadwell A (2017). Visualisation of upper limb activity using spirals: A new approach to the assessment of daily prosthesis usage. Prosthet. Orthot. Int..

[7] Chadwell, A., Kenney, L., Thies, S., Galpin, A. & Head, J. The Reality of Myoelectric Prostheses: Understanding What Makes These Devices Difficult for Some Users toControl. *Frontiers in Neurorobotic*s **10**10.3389/fnbot.2016.00007 (2016). 10.3389/fnbot.2016.00007 PMC4992705 27597823

[8] Bailey RR, Klaesner JW, Lang CE (2015). Quantifying real-world upper-limb activity in nondisabled adults and adults with chronic stroke. Neurorehabilitation and Neural Repair.

[9] Hill W (2009). Upper limb prosthetic outcome measures (ULPOM): A working group and their findings. JPO Journal of Prosthetics and Orthotics.

[10] Wright V (2009). Prosthetic outcome measures for use with upper limb amputees: A systematic review of the peer-reviewed literature, 1970 to 2009. JPO: Journal of Prosthetics and Orthotics.

[11] Lindner HYN, Nätterlund BS, Hermansson LMN (2010). Upper limb prosthetic outcome measures: Review and content comparison based on international classification of functioning, disability and health. Prosthet. Orthot. Int..

[12] Bouwsema H, Kyberd PJ, Hill W, van der Sluis CK, Bongers RM (2012). Determining skill level in myoelectric prosthesis use with multiple outcome measures. Journal of rehabilitation research and development.

[13] Bouwsema H, der Sluis CK (2010). v. & Bongers, R. M. Movement characteristics of upper extremity prostheses during basic goal-directed tasks. Clinical Biomechanics.

[14] Gallagher, P. *Trinity Amputation and Prosthesis Experience Scales - Revised (TAPES-R)* http://psychoprosthetics.ie/assets/TAPES_2011_Sept_2011.pdf (2011).

[15] Cain KL, Conway TL, Adams MA, Husak LE, Sallis JF (2013). Comparison of older and newer generations of ActiGraph accelerometers with the normal filter and the low frequency extension. International Journal of Behavioral Nutrition and Physical Activity.

[16] A Corp. AG White Paper: What is a count? (49E Chase Street Pensacola FL 32502, 2015).

[17] Philip BA, Frey SH (2014). Compensatory Changes Accompanying Chronic Forced Use of the Nondominant Hand by Unilateral Amputees. The Journal of Neuroscience.

[18] Major MJ, Stine RL, Heckathorne CW, Fatone S, Gard SA (2014). Comparison of range-of-motion and variability in upper body movements between transradial prosthesis users and able-bodied controls when executing goal-oriented tasks. Journal of NeuroEngineering and Rehabilitation.

[19] Thies SB (2017). Skill assessment in upper limb myoelectric prosthesis users: Validation of a clinically feasible method for characterising upper limb temporal and amplitude variability during the performance of functional tasks. Medical Engineering and Physics.

[20] Thies S (2009). Movement variability in stroke patients and controls performing two upper limb functional tasks: a new assessment methodology. Journal of NeuroEngineering and Rehabilitation.

[21] Sobuh M (2014). Visuomotor behaviours when using a myoelectric prosthesis. Journal of NeuroEngineering and Rehabilitation.

[22] Parr, J. V. V., Vine, S. J., Harrison, N. R. & Wood, G. Examining the Spatiotemporal Disruption to Gaze When Using a Myoelectric Prosthetic Hand. *Journal of Motor Behavior* 1-10 10.1080/00222895.2017.1363703 (2017). 10.1080/00222895.2017.1363703 28925815

[23] Bailey RR, Lang CE (2014). Upper extremity activity in adults: Referent values using accelerometry. Journal of rehabilitation research and development.

[24] Spiers, A., Resnik, L. & Dollar, A. Classifying and quantifying unilateral prosthesis use in home environments to inform device and treatment design. *Myoelectric Control Symposium* 95 ID#47 (2017).

[25] Zhou, S.-M. *et al*. Classification of accelerometer wear and non-wear events in seconds for monitoring free-living physical activity. *BMJ Open* **5**10.1136/bmjopen-2014-007447 (2015). 10.1136/bmjopen-2014-007447 PMC4431141 25968000

[26] Kyberd PJ (2009). Case studies to demonstrate the range of applications of the Southampton Hand Assessment Procedure. Brit J Occup Ther.

[27] Kyberd P, Hussaini A, Maillet G (2018). Characterisation of the Clothespin Relocation Test as a functional assessment tool. Journal of Rehabilitation and Assistive Technologies Engineering.

