# Supplementary material for: Upper limb activity in myoelectric prosthesis users is biased towards the intact limb and appears unrelated to goal-directed task performance
Source: Sci Rep. 2018 Jul 23;8:11084. doi: 10.1038/s41598-018-29503-6 (PMC6056489; doi:10.1038/s41598-018-29503-6)
Supplement: Supplementary file 1 — Supplementary Material [file 41598_2018_29503_MOESM1_ESM.docx]

SUPPLEMENTARY MATERIAL: Upper limb activity in myoelectric prosthesis users is biased towards the intact limb and appears unrelated to goal-directed task performance

**Authors:** Chadwell A.^1^*, Kenney L.^1^, Granat MH.^1^, Thies S.^1^, Head J.^1^, Galpin A.^1^, Baker R.^2^, Kulkarni J.^3^

**Author affiliations:**

1. Centre for Health Sciences Research, University of Salford
2. Salford Business School, University of Salford
3. University Hospital of South Manchester NHS Foundation Trust

**Author contact information:** *a.e.a.chadwell1@salford.ac.uk

## Background

This study uses wrist-worn activity monitoring sensors to record the upper limb activity of prosthesis users and anatomically intact adults. To accurately analyse upper limb activity, it is important to determine the periods when the monitors are, and are not, worn.

Anatomically intact participants were instructed to remove the monitors if they were likely to get wet. Therefore it can be assumed that when either monitor was removed (for example, to shower), both would be removed (resulting in no activity counts being recorded on either monitor VM=0). These periods where no activity is recorded on either arm are excluded as part of the data analysis. The algorithm described below was therefore only applied to data from the prosthesis users.

Prosthesis users are likely to remove the prosthesis for periods during the day, leaving the second monitor on the intact wrist. Without further processing, the data from these periods would present incorrectly as unilateral activity on the anatomically intact side, thereby biasing the results. Therefore, a method is needed to identify periods when the prosthesis and the attached monitor were removed. We refer to these periods as “***prosthesis non-wear***”, although we are currently unable to differentiate between removal of the prosthesis, and removal of the prosthesis monitor from the wrist of the prosthesis.

As participants were instructed to leave the monitor on the wrist of the prosthesis at all times, it can be assumed that when showering etc. the myoelectric prosthesis itself would have been removed. Furthermore, as there would be no discomfort associated with wearing the monitor on the wrist of the prosthesis, it is reasonable to assume that participants complied with this instruction.

“***Prosthesis wear time***” therefore refers to the times when both the prosthesis *and* the monitor on the prosthetic ‘wrist’ were worn; “***prosthesis non-wear***” was calculated based on the activity counts recorded on the prosthesis worn monitor.

For the results presented in the main body of the paper, the algorithm presented here was only used to remove the periods “***prosthesis non-wear***” in order to avoid the potential bias discussed earlier.

To differentiate between self-reported wear times and wear times calculated using the algorithm the suffixes “***(SR)***” and “***(C)***” are used.

## Aims

No standardised method exists to distinguish wrist worn accelerometer wear from non-wear ^[1]^. In the pilot stages of this work, “***prosthesis non-wear***” periods were removed through a combination of automated event detection, diary data, and visual inspection ^[2]^. Here we report on the development of a fully automated method of “***prosthesis non-wear***” detection.

## Proposed algorithm for the detection of prosthesis non-wear

The algorithm has been developed on the assumption that prolonged periods of activity recorded on the prosthesis worn monitor constitute “***prosthesis wear***”, and that prolonged periods of inactivity correspond to “***prosthesis non-wear***”. As noted in our previous work ^[2]^, occasionally isolated spikes may be seen in the Vector Magnitude data which may not correspond to “***prosthesis wear***”, and similarly, short periods of inactivity may occur during “***prosthesis wear***” periods. The algorithm is therefore designed to inspect surrounding data points during the classification of each epoch.

Data were collected using Actigraph activity monitoring sensors from the GT3X range (GT3X+, wGT3X, wGT3X-BT) and downloaded using the Actilife 6 software where they were filtered using the low frequency extension filter (proprietary ^[3]^) and grouped into 60s epochs. For each epoch, acceleration data were converted into activity counts (proprietary ^[4]^), and summed across the three axes to generate Vector Magnitudes of the activity counts ($VM=\sqrt{x^{2}+y^{2}+z^{2}}$). The Vector Magnitude values were imported into Matlab for the removal of non-wear periods.

Each epoch was classified as either wear or non-wear according to the steps below (see also **Figure 1**).

**Step 1:** For the first epoch (minute 1), if the Vector Magnitude was equal to zero (no counts recorded) the epoch was classified as non-wear, otherwise it was classified as wear.

**Step 2:** Working from the second epoch (minute 2) to the last (minute 10080), each epoch was compared to the previous epoch, if no counts were recorded (VM=0) and the previous epoch had been classified as non-wear, it was assumed the monitor was still not being worn and this epoch was also classified as non-wear. Similarly if counts were recorded, and the previous epoch had been classified as wear, it was assumed the monitor was still being worn and this epoch was also classified as wear. If the epoch was identified as a possible transition between wear and non-wear (e.g. VM=0 but previous epoch=wear; or VM>0 but previous epoch=non-wear) the epoch was assessed according to Step 3.

**Step 3:** Transitions between wear and non-wear periods were more complex to detect. Step 3 aims to avoid misclassification based on isolated spikes of data or short periods of inactivity.

A possible transition from non-wear to wear: Where activity was recorded for the epoch under inspection (VM>0), but the previous epoch had been classified as non-wear, the following checks were used to establish whether the current epoch should be categorised as wear.

1. If the Vector Magnitude of the epoch under inspection was greater than 15^[[1]](#footnote-1)^, and the monitors continued to show activity over the subsequent 20 minutes^[[2]](#footnote-2)^, demonstrated by no-more than 5 consecutive minutes of VM≤15, the epoch was classified as wear.
2. Otherwise, if the Vector Magnitude of the epoch under inspection was less than or equal to 15, or the monitors showed prolonged inactivity over the subsequent 20 minutes, demonstrated by more than 5 consecutive minutes of VM≤15, the epoch was classified as non-wear.

A transition from wear to non-wear: Where no activity was recorded for the epoch under inspection (VM=0), but the previous epoch had been classified as wear, the following checks were used to establish whether the current epoch should be categorised as non-wear.

1. If the monitors continued to show inactivity over the subsequent 20 minutes^[[3]](#footnote-3)^, demonstrated by no-more than 5 consecutive minutes of VM>15, the epoch was classified as non-wear.
2. Otherwise, if the monitors showed prolonged periods of activity over the subsequent 20 minutes, demonstrated by more than 5 consecutive minutes of VM>15, the epoch was classified as wear.


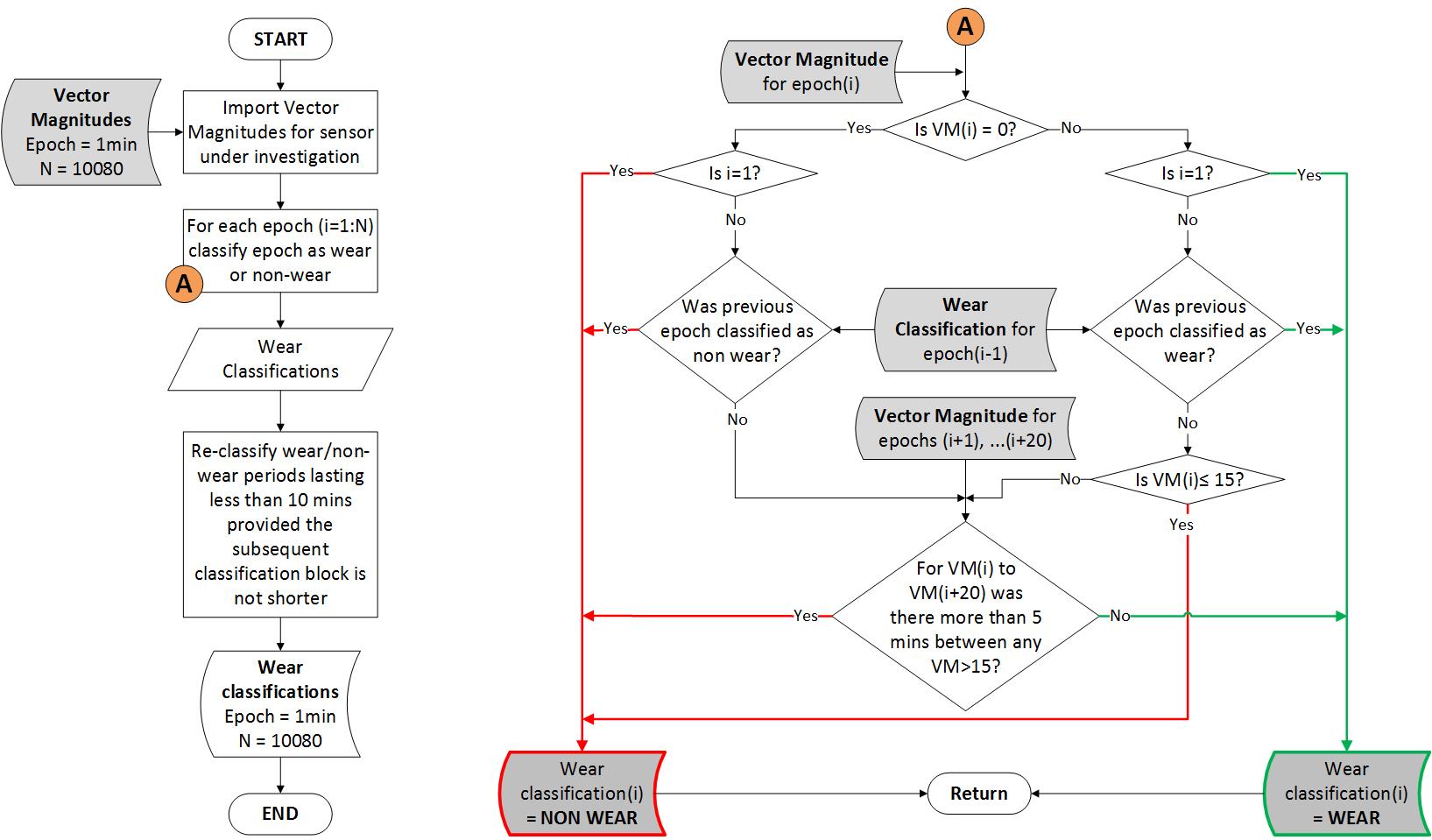


Figure 1. Automated non-wear algorithm.

**Step 4:** Initial testing of the algorithm suggested that some epochs had been misclassified; this occurred where two ‘isolated’ spikes occurred within 5 minutes of each other during a non-wear period resulting in an incorrect classification of wear. This was remedied by undertaking a second classification phase; periods of wear/non-wear lasting for less than 10 minutes were re-classified unless they were immediately followed by a shorter block of wear/non-wear.

## Comparison to self-reported prosthesis non-wear

Participants were asked to complete a wear diary to assist with the development of the non-wear algorithm. Nineteen participants returned the wear diary; the self-reported prosthesis and/or monitor wear times were incomplete for five of these participants. For the remaining fourteen participants “***prosthesis wear time (C)***” calculated using the algorithm was plotted against the self-reported “***prosthesis wear time (SR)***”. The discrepancy between the measures was highlighted.

Over the 7 days “***Prosthesis wear time (C)***” was on average (median) 4.4 hours shorter than “***Prosthesis wear time (SR)***” (min = 52.6 hours shorter, Q1 = 9.5 hours shorter, Q3 = 0.8 hours longer, max = 6.3 hours longer) (**Figure 2**).


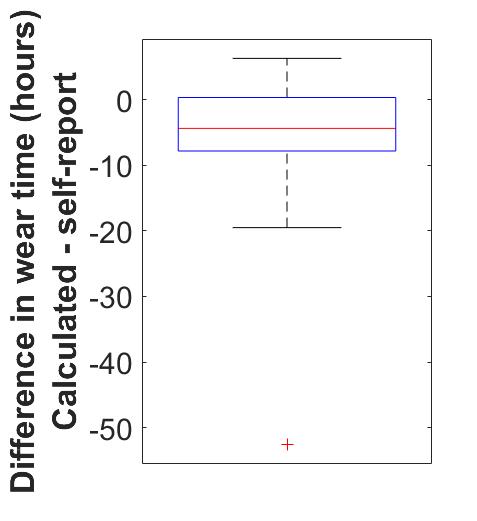


Figure 2. Box plot representing the difference between the “*prosthesis wear time (C)*” and “*prosthesis wear time (SR)*” for 14 participants.

There are some limitations to the algorithm, one participant self-reported to remove the prosthesis when driving each day; and this can be seen in a reduction in the Vector Magnitude during these periods (**Figure 3A**); these periods were not detected as “***prosthesis non-wear (C)***” by the algorithm. Similarly periods where the prosthesis or monitors were removed for less than 20 minutes (e.g. a quick shower) (**Figure 3B**) were not detected as non-wear “***prosthesis non-wear (C)***” using this algorithm. Further work would be needed to ensure that this algorithm was robust to all situations, however this was outside of the scope of this study. For the majority of participants, the algorithm appeared to calculate “***prosthesis non-wear***” more accurately than self-reported (**Figure 3C**).

## Checking the performance of the algorithm to detect monitor removal on anatomically intact subjects

The data presented above suggests that on average the algorithm was able to accurately detect “***prosthesis non-wear***”; although there were some periods that were self-reported as “***prosthesis wear***”, which the algorithm allocated as “***prosthesis non-wear***”. To further evaluate the ability of the algorithm to detect non-wear periods, the same algorithm was used to analyse the “***anatomical monitor non-wear***” for the cohort of anatomically intact participants. The algorithm was used to detect the removal of the monitor worn on the dominant wrist.

Figure 3. Each figure presents the Vector Magnitude data recorded by the monitor worn on the wrist of the prosthesis over 24 hours. The bars below allow comparison of the “*prosthesis wear time (C)*” (red), and “*prosthesis wear time (SR)*” (green). The discrepancy between the two measures is shown in blue. The magenta arrows indicate specific points discussed in the main text: (A) the participant self-reported to remove the prosthesis when driving, (B) the participant self-reported to remove the prosthesis for 14 minutes, and (C) the participant self-reported to remove the prosthesis at midday.

All twenty anatomically intact participants involved in the study returned completed diaries. Data was plotted and the discrepancy between the “***anatomical monitor wear time (SR)***” and the “***anatomical monitor wear time (C)***” was highlighted. Visual inspection suggested that although the algorithm was consistent in the detection of “***anatomical monitor wear time***” during the daytime, whilst the person was asleep, the algorithm was not very accurate (see **Figure 4**). For the purposes of this study, it was not important that the non-wear algorithm was able to accurately detect the monitor wear status during the times the person was asleep; the self-reported sleep times were therefore excluded from the following analysis.

Figure 4. Example plot displaying 24 hours of data recorded from one of the monitors worn by an anatomically intact participant. During the times the participant self-reported to be awake (magenta) the “*anatomical monitor wear time*” calculated using the algorithm (red) matched the self-reported “*anatomical monitor wear time*” (green). Whilst the participant was asleep, the algorithm was less accurate.

During the times the participants self-reported to be awake (over the 7 days), the “***anatomical monitor wear time (C)***” was on average (median) 1 minute shorter than the “***anatomical monitor wear time (SR)***” (min = 8.0 hours shorter, Q1 = 1.7 hours shorter, Q3 = 0.7 hours longer, max = 1.5 hours longer) (**Figure 5**).

For the three outliers shown in **Figure 5**, the large discrepancy between the “***anatomical monitor wear time (SR)***” and “***anatomical monitor wear time (C)***” could possibly be explained by: (1) lying in bed in the morning (one participant self-reported to wake up to 3 hours before the monitor detected large amounts of movement), (2) sitting still for long periods of time, or (3) removing the monitor without reporting its removal (See **Figure 6** for examples of large discrepancies).

## Conclusion

Detection of “***prosthesis non-wear***” is a complex task. The prosthesis may be carried or transported resulting in movement detection on the activity monitor; furthermore, detection of short periods of prosthesis removal is difficult without misclassifying periods where the person may have been sat still (for example watching TV). For the majority of participants, visual inspection of the plots suggested that the automated non-wear algorithm was more accurate than the self-report data. Self-report generally overestimated the wear time.

In future, a more complex algorithm for the detection of “***prosthesis non-wear***” would be beneficial.


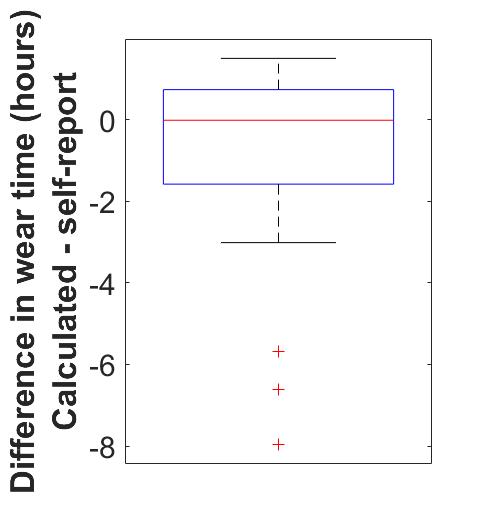


Figure 5. Box plot representing the difference between the “*anatomical monitor wear time (C)*” and “*anatomical monitor wear time (SR)*” for 20 anatomically intact participants.

Figure 6. These three plots demonstrate some of the large discrepancies between “*anatomical monitor wear time (SR)*”, and “*anatomical monitor wear time (C)*”. The Vector Magnitude data (over 24 hours) is presented from the monitor worn on the dominant wrist. The bars below allow comparison of the “*anatomical monitor wear time (C)*” (red), and “*anatomical monitor wear time (SR)*” (green). The discrepancy between the two measures is shown in blue. The magenta bar signifies the time the participant self-reported to be awake. Possible explanations for the large discrepancies, marked with the orange arrows could be: (A) the participant remained in bed, (B) the participant sat very still, and (C) incorrect self-report, it is possible the monitor was not actually worn.

1. Zhou, S.-M., et al., *Classification of accelerometer wear and non-wear events in seconds for monitoring free-living physical activity.* BMJ Open, 2015. **5**(5).

2. Chadwell, A., et al., *The Reality of Myoelectric Prostheses: Understanding What Makes These Devices Difficult for Some Users to Control.* Frontiers in Neurorobotics, 2016. **10**(7).

3. Cain, K.L., et al., *Comparison of older and newer generations of ActiGraph accelerometers with the normal filter and the low frequency extension.* International Journal of Behavioral Nutrition and Physical Activity, 2013. **10**(1): p. 51.

4. Actigraph Corp, *ActiGraph White Paper: What is a count?* 2015: 49E Chase Street, Pensacola, FL 32502.

1. This threshold was chosen through visual inspection of the data spikes generated by picking up the sensors/prosthetic arm. [↑](#footnote-ref-1)
2. This ensured that isolated spikes of activity within a non-wear period were not incorrectly coded as wear. [↑](#footnote-ref-2)
3. This ensured that short periods of inactivity within a wear period were not incorrectly coded as non-wear. [↑](#footnote-ref-3)
